# Regulation of Acetylation States by Nutrients in the Inhibition of Vascular Inflammation and Atherosclerosis

**DOI:** 10.3390/ijms24119338

**Published:** 2023-05-26

**Authors:** Hyunju Kang

**Affiliations:** Department of Food and Nutrition, Keimyung University, Daegu 42601, Republic of Korea; hyunjukang@kmu.ac.kr

**Keywords:** nutrients, acetylation state, vascular inflammation, atherosclerosis

## Abstract

Atherosclerosis (AS) is a chronic metabolic disorder and primary cause of cardiovascular diseases, resulting in substantial morbidity and mortality worldwide. Initiated by endothelial cell stimulation, AS is characterized by arterial inflammation, lipid deposition, foam cell formation, and plaque development. Nutrients such as carotenoids, polyphenols, and vitamins can prevent the atherosclerotic process by modulating inflammation and metabolic disorders through the regulation of gene acetylation states mediated with histone deacetylases (HDACs). Nutrients can regulate AS-related epigenetic states via sirtuins (SIRTs) activation, specifically SIRT1 and SIRT3. Nutrient-driven alterations in the redox state and gene modulation in AS progression are linked to their protein deacetylating, anti-inflammatory, and antioxidant properties. Nutrients can also inhibit advanced oxidation protein product formation, reducing arterial intima-media thickness epigenetically. Nonetheless, knowledge gaps remain when it comes to understanding effective AS prevention through epigenetic regulation by nutrients. This work reviews and confirms the underlying mechanisms by which nutrients prevent arterial inflammation and AS, focusing on the epigenetic pathways that modify histones and non-histone proteins by regulating redox and acetylation states through HDACs such as SIRTs. These findings may serve as a foundation for developing potential therapeutic agents to prevent AS and cardiovascular diseases by employing nutrients based on epigenetic regulation.

## 1. Introduction

Atherosclerosis (AS), a chronic inflammatory ailment of the arterial system, pertains to the intima of medium or large arteries, associated with dyslipidemia as well as alterations in the arterial wall composition [1]. The inflammatory process leads to vascular dysfunction by forming atherosclerotic plaques due to vascular inflammation (VI). The initial pathogenic event in AS is the dysfunction of endothelial cells (ECs), resulting from the disturbed vascular flow. The excessive production of reactive oxygen species (ROS), by enzymatic pathways including uncoupled nitric oxide synthase (NOS) and NADPH oxidases (NOXs), leads to dysfunction of ECs and vascular smooth muscle cells (VSMCs), and inflammation, leading to AS. AS has been accompanied by accumulated modified lipids, foam cell formation, vascular wall fibroblasts, apoptosis, and calcification [2].

Epigenetic therapy, mediated by histone modifications, has proven effective in regulating acetylation state through nutrient administration. Pharmacological modulation of acetylation state by activating class III histone deacetylases (HDACs), such as sirtuin 1 and sirtuin 3 (SIRT1 and SIRT3) with small natural nutrient molecules, has been shown to protect against inflammation and metabolic dysfunction associated with VI and AS [3]. Conversely, inhibition of class I and II HDACs by nutrients has been suggested to ameliorate inflammatory and metabolic responses. Dynamic epigenetic regulation governing differentiation and activation of monocytes and macrophages, may affect inflammatory and oxidative pathways associated with AS [4]. Epigenetic regulation of enzymes that control excessive ROS production by HDACs, such as SIRTs, has been proposed to inhibit the progression of AS by suppressing EC dysfunction and inflammation [3,5]. SIRT1 is known to protect against various oxidative stress and aging-induced EC dysfunction and inflammation. This protective effect is achieved through deacetylation, which subsequently enhances the ability of cellular components to inhibit inflammatory and oxidative responses [6]. Foam cell formation by VSMCs, monocytes, or macrophages can initiate the development of AS [7]. The process of reverse cholesterol transport, from peripheral cells to the liver, can effectively eliminate excess cholesterol from arterial cells, thus preventing foam cell formation from VSMCs and macrophages [8]. Hepatic lipid accumulation can be reduced by SIRT1 by activating AMP-activated protein kinase (AMPK) [9] and peroxisome proliferator-activated receptor α (PPARα) signaling [3].

Long-term epigenetic remodeling of innate immune cells induced by pro-atherogenic stimuli, including low-density lipoprotein (LDL) cholesterol and oxidized LDL, can trigger continuous activation even after the stimuli are removed [10]. In other words, innate immune cells such as monocytes, macrophages, or natural killer cells can acquire non-specific memories by epigenetic modulation that alters their response to subsequent stimuli, based on this trained immune system [11]. Dietary stress induced by alcohol, high triglyceride, or high cholesterol diets has been proven to impede the activation of SIRT1 and SIRT3, which in turn promotes VI and the development of atherosclerotic plaques [12]. Cumulative studies showed the epigenetic control of VI and AS by DNA methylation [4]; however, relatively little attention has been paid to pathways leading to nutrient-related VI and AS by regulating the acetylation state. Given that dietary stress stimulates the histone acetyltransferases (HATs) to enhance the transcription of AS-related genes, regulating HDACs activity through the control of the acetylation state may offer potential roles for nutrients in addressing the pathogenesis leading to VI and AS. The present study examined the still emerging and controversial epigenetic function of nutrients in mitigating VI and AS through the regulation of histone acetylation state.

## 2. Epigenetic Regulation of VI and AS

Epigenetic regulations of histone acetylation state are composed of three steps: detection, acetylation, and deacetylation [13]. Histone acetylation is initiated by detecting specific signaling in the bromodomain and extra-terminal domain family of proteins, called readers, which bind acetyl–lysine and link to a series of covalent histone modifications. Histone acetylation is mediated by HATs called writers, while deacetylation is mediated by HDACs called erasers [14]. The coordinated epigenetic action of readers, writers, and erasers with the help of various related enzymes leads to fine-tuned gene expression by relaxing or condensing chromatin structure to target gene promoters.

### 2.1. Factors Affecting VI and AS

Vascular ECs facilitate a thin layer in the inner wall of blood vessels to regulate their responses to diverse stimuli, including oxidative stress, inflammation, and shear stress [15]. Endothelial dysfunction, characterized by increased endothelial vascular permeability and the migration and proliferation of ECs, serves as an indicator of AS [2]. Risk factors for AS include hypertension, smoking, hyperlipidemia, insulin resistance, obesity, and metabolic dysfunction [1]. Substantial alterations in blood vessel morphology, such as bifurcation, flexion, and narrowing of arteries, contribute to the increased incidence of AS [2]. Pathophysiological processes of AS are directly associated with VI, which can be influenced by various stresses and dysfunctions, including an imbalance of blood glucose and plasma lipid in the artery [15]. To avoid VI and prevent AS, it is essential to maintain the homeostasis of blood glucose and plasma lipid, which can be regulated by the conditions of vascular ECs. In particular, regulating detrimental events such as inflammation, shear stress, and oxidative stress is a crucial factor for maintaining the homeostasis of blood vessels [16]. Endothelial dysfunctions, resulting in enhanced vascular permeability and the migration and proliferation of ECs, trigger AS [17].

Vascular dysfunction and features of AS can be attributed to both physical and chemical factors. The physical factors are ascribed to the disturbing forces of blood flow in arteries, including fluid shear stress, tensile forces, and hydrostatic pressure forces [18]. Disturbed blood flow in the curvature and bifurcation regions can promote inflammation and AS by activating activator protein 1 (AP-1), nuclear factor-κB (NF-κB), and protein kinase C (PKC) [19]. The chemical risk factors contributing to AS involve inflammation, oxidative stress, and bioavailability of nitric oxide (NO), which plays a regulatory role in managing the redox state, NF-κB, p53, and endothelial NOS (eNOS) pathways [20]. Inflammatory molecules such as interleukin-1 (IL-1), IL-6, cyclooxygenase-2 (COX-2), matrix metalloproteinase-2 (MMP-2), and MMP-9, adhesion molecules such as intercellular adhesion molecule 1 (ICAM-1) and vascular cell adhesion molecule 1 (VCAM-1), and angiogenic growth factors can induce inflammation and EC dysfunction, resulting in vascular remodeling and angiogenesis [10].

From an immunological view, inflammatory molecules play a crucial role in recruiting and promoting monocytes to transmigrate across the endothelial monolayer into the sub-intima of the vessel wall, where they proliferate and differentiate into macrophages and foam cells by taking up lipoproteins [21]. The progressive death of these foam cells and macrophages, along with the release of lipid-filled contents and tissue factors lead to the formation of a lipid-rich necrotic core, which destabilizes plaques [21]. Meanwhile, VSMCs migrate from the medial layer and accumulate in the intima, where they form the fibrous cap over the lesion by secreting interstitial collagen and elastin. However, cytokines and matrix-degrading proteases secreted by macrophages and lymphocytes, such as collagenase, gelatinase, stromelysin, and cathepsin, weaken the atherosclerotic plaques [22], eventually breaking the thin fibrous caps and plaques. The exposed pro-coagulant materials released into the blood trigger thrombosis, which can impede blood flow and result in acute stenosis of the arteries [23].

The key features of AS have been identified as a major contributor to cardiovascular disease, increasing the risk of acute myocardial infarction and stroke. Atherosclerotic lesions develop in the intima of arteries due to chronic inflammation, resulting from lipid accumulation with deposited macrophages and T cells. This process leads to increased atherosclerotic plaque growth and triggers blood clot formation [24]. Long-term vascular damage with a chronic inflammatory response, resulting from factors such as high shear stress, free radicals, elevated cholesterol levels, and oxidized LDL, contributes to the initiation of atherosclerotic plaque formation [25].

### 2.2. Regulation of VI and AS

**Figure 1:**
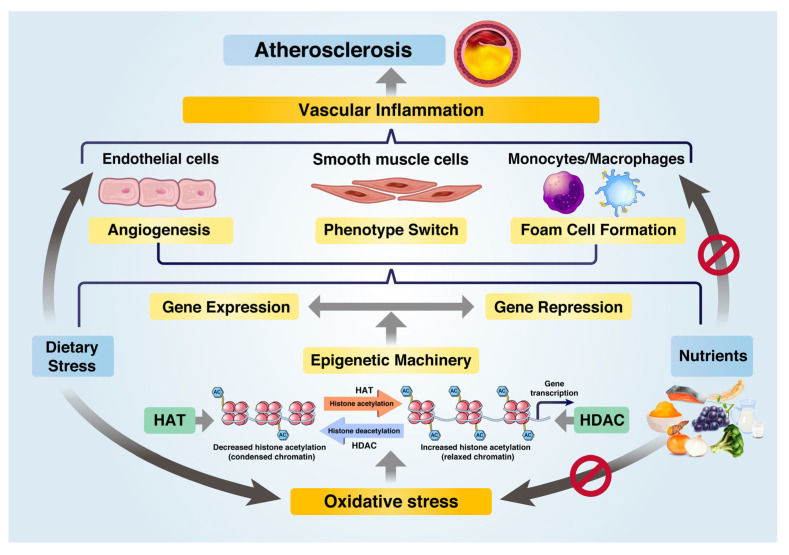
Role of nutrients in preventing atherosclerosis progression by epigenetic regulation of gene expression under dietary stress. Under dietary stress, HATs induce oxidative stress and inflammatory gene expression via epigenetic machinery. Nutrients serve as epigenetic regulators, reducing oxidative stress and repressing inflammatory gene expression through HDACs such as SIRT1 and SIRT3. Nutrients inhibit dietary-induced inflammation and metabolic dysfunctions in ECs, VSMCs, and macrophages, thereby preventing atherosclerosis progression.

It has been demonstrated that AS is an epigenetic disorder [14], indicating that epigenetic regulation can be a promising approach to prevent AS [22]. Dietary stress leads to metabolic disorders, such as dysfunctions in glucose and lipid metabolism, which can be ameliorated through epigenetic regulation by utilizing HDACs. The regulation of acetylation states of enzymes and genes involved in oxidative metabolism plays a crucial role in controlling blood glucose and plasma lipid levels, and subsequently, affecting the progression of VI and AS. Figure 1 presents an overview of the epigenetic regulation schemes of nutrients that can inhibit AS.

Evidence suggests that class II HDACs may contribute to the progression of AS, but SIRT1 has a preventive role in it [3]. SIRT1 can inhibit AS-related chemical risk factors, including inflammation, oxidative stress, and NO bioavailability, by controlling redox state, inhibiting NF-κB and p53, and activating eNOS [26]. During the initial stages of AS, fat-driven deposits at the vascular walls induce neointima formation by hypercholesterolemia, resulting in hypoxia and induction of hypoxia-inducible factor (HIF)-1, stimulating neovascularization and developing atherosclerotic plaque [27]. SIRT1 can inhibit neointima formation by suppressing HIF-1α expression through deacetylation in hypoxic VSMCs [28]. The structure and function of the arterial wall is determined by the phenotypes of VSMCs, such as their contractile and dedifferentiated state, which play a crucial role in response to atherosclerotic factors. The transition from a contractile to a dedifferentiated state has been shown to have protective effects against AS [29]. During the progression of AS, VSMCs with excessive cholesterol migrate into the intima, where they become modified to an inflammatory phenotype and become the predominant cell type in early arterial intimal thickening [30]. As VSMCs are an essential source of foam cells along with macrophages [31], the epigenetic restoration of VSMC functions reliant on SIRT1 may effectively hinder AS development.

The properties of SIRT1 as an anti-AS can be attributed to its capacity to modulate inflammatory responses and cholesterol metabolism. Specifically, SIRT1 has been shown to suppress the expression of pro-inflammatory mediators such as tumor necrosis factor α (TNFα), IL-6, NF-κB, monocyte chemoattractant protein-1 (MCP-1), ICAM-1, and VCAM-1. Furthermore, it can reduce levels of serum free fatty acids, blood glucose, triglyceride, and total cholesterol, and inhibit inflammatory cell infiltration in atherosclerotic plaques [29]. Given the ability of SIRT1 to regulate common pathways leading to both AS and metabolic disease, it is suggested that the inhibition of AS may be achieved through similar mechanisms employed in metabolic disease prevention, partly attributed to SIRT1 [32].

Foam cell formation can trigger the progression of AS, but this can be prevented by promoting reverse cholesterol transport to remove excess cholesterol from arterial cells, thereby inhibiting foam cell formation in VSMCs and macrophages [8]. The liver can discard accumulated lipids by increasing fatty acid oxidation and ketogenesis, inhibiting hepatic steatosis and inflammation induced by high-fat diets through SIRT1 activation [33]. By suppressing the NF-κB signaling pathway, SIRT1 can reduce the expression of lectin-like oxidized LDL receptor-1 (LOX-1), thereby inhibiting the formation of foam cells in macrophages and monocytes [34]. However, dietary stress induced by alcohol or high-triglyceride or high-cholesterol diet can inhibit the abilities of SIRT1, preventing reverse cholesterol transport and enhancing inflammation, which reduces the deletion of foam cells from atherosclerotic plaques [35].

SIRT1 has been shown to control autophagy by generating a molecular complex and by deacetylating proteins involved in the autophagy process, such as autophagy related 5 (ATG5), ATG7, and ATG8 [36]. This SIRT1-mediated autophagy has been found to protect against foam cell formation in VSMCs treated with oxidized LDL [37]. Furthermore, the toxic effects of oxidized LDL can induce cell apoptosis in atherosclerotic areas, including human smooth muscle cells [38]. The accumulation of apoptotic cells in the vascular wall can lead to plaque erosion, promoting local platelet aggregation and thrombosis, which in turn can prevent the progression of AS [39]. Thus, autophagy appears to be another mechanism by which SIRT1 exerts its anti-atherosclerotic effects.

Several studies have verified that SIRT’s deacetylation capability can initiate a variety of antioxidant actions, including superoxide dismutase 2 (SOD2) and catalase, which aid in maintaining the oxidant/antioxidant equilibrium and mitigating vascular endothelial oxidative stress [3]. Upregulating the SIRT3/SOD2 signaling pathway can facilitate the capacity of endothelial progenitor cells to re-endothelialize and conserve mitochondrial redox balance [40]. In rats subjected to a high-fat diet, SIRT1 expression diminished, while serum levels of total cholesterol, triglyceride, and LDL cholesterol considerably increased, coupled with a marked reduction in high-density lipoprotein cholesterol levels [41]. This implies that a prolonged high-fat diet may negatively impact SIRT3 functionality. Since the SIRT3 gene promoter region is identified by estrogen-associated receptor (EAR), PPARγ coactivator 1α (PGC-1α) facilitates the binding of EAR to the sequence motif in the SIRT3 promoter, thereby promoting SIRT3 expression [42]. SIRT3 can amplify the activity of long-chain acyl CoA dehydrogenase, a crucial enzyme in lipid metabolism, through direct deacetylation and promoting fatty acid oxidation [43]. SIRT3 is known to reduce lipotoxicity across various tissues, such as liver. Ectopic fat deposition, as observed in the liver and epicardial fat, may elevate the risk of AS [44]. It has been shown that SIRT3 can deacetylate and activate liver kinase B1, thereby stimulating the AMPK signaling pathway and facilitating lipid autophagy [42]. Overexpression of SIRT3 can suppress lipid accumulation in macrophages and diminish the formation of foam cells through isocitrate dehydrogenase 2 (IDH2) deacetylation [45].

Evidence has shown that SIRT3 plays a role in regulating EC metabolism through deacetylation, increasing NADPH levels, and subsequently raising the ratio of reduced glutathione in mitochondria [46], to protect ECs from oxidative stress. ECs primarily acquire energy from glycolysis rather than oxidative phosphorylation [47]. Deficiency of SIRT3 results in increased acetylation and diminished activity of endothelial glycolytic enzyme 6-phosphofructo-2-kinase/fructose-2,6-biphosphatase 3 (PFKFB3), leading to excessive mitochondrial respiration and ROS production, ultimately reducing glycolysis and angiogenesis [48]. Moreover, in SIRT3-deficient ECs, the expression of hypoxia-induced HIF-2, vascular endothelial growth factor (VEGF), and angiopoietin 1 (Ang1) is activated due to increased mitochondrial ROS, impacting vascular endothelial permeability and systolic function [48]. These findings imply that SIRT3 is crucial for vascular EC homeostasis and presents a promising target for preventing AS.

## 3. Roles of Nutrients to Inhibit VI and AS

**Figure 2:**
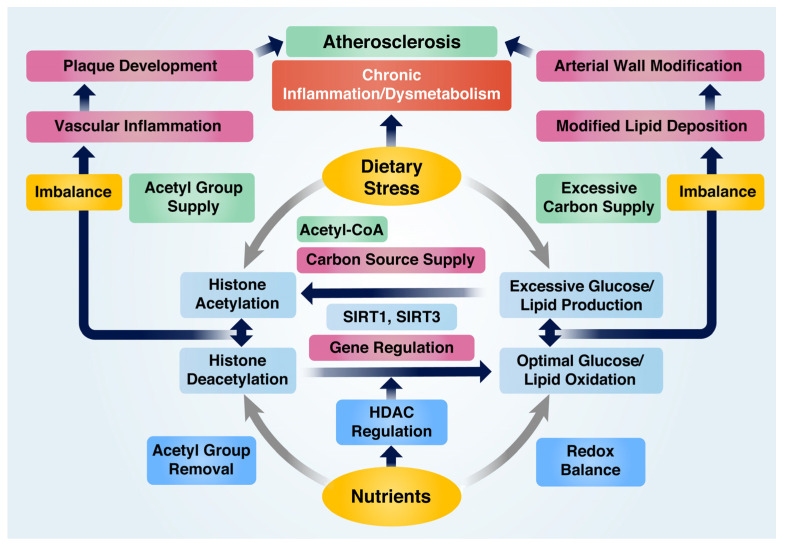
Nutrient-mediated prevention of atherosclerosis by modulating redox and acetylation balance under inflammatory and abnormal metabolic conditions. Dietary stress contributes to histone acetylation by supplying acetyl groups, whereas nutrients facilitate deacetylation. An imbalance between these processes triggers pro-inflammatory gene expression and chronic inflammation. Dietary stress generates an excessive carbon source for the production of extra glucose and lipids, leading to increased carbon supply for histone acetylation and resulting in hyperglycemia and hyperlipidemia. On the other hand, nutrients regulate genes associated with glucose and lipid oxidation and maintain redox balance. Disruptions in glucose and lipid metabolism cause chronic metabolic dysfunction, but nutrients can modulate chronic inflammation and metabolic dysfunction to prevent atherosclerosis.

The pathological pathways leading to VI and AS have primarily been attributed to imbalance in redox and acetylation states, depending on the micro-environmental conditions. The ability of nutrients to inhibit VI and AS can be categorized into two main aspects: their involvement in managing oxidative stress that leads to inflammation and their function in altering histone acetylation states. Core histone proteins, such as histones H3 and H4, are targeted for acetylation by HATs or deacetylation by HDACs [49]. Epigenetic transcription or repression of genes resulting from altered histone acetylation states depends on the modification site of the residues and chromatin remodeling features, which regulate chromatin status as either condensed or relaxed [50]. Dietary stress is known to enhance the expression of inflammation-associated genes through HATs, such as p300 and cAMP-response element binding protein (CBP), while nutrient-derived HDACs exhibit opposite effects [51]. In particular, SIRTs such as SIRT1 and SIRT3 are crucial HDACs induced by various nutrients that participate in essential biological processes through the deacetylation of histones at H3K9, H3K18, and H3K56 [52]. In addition, they deacetylate non-histone protein substrates associated with regulating inflammation and metabolism, such as p53, liver X receptor (LXR), PGC-1α, NF-κB, and forkhead box O protein (FOXO) [52]. The role of nutrients in preventing AS by regulating the balance of redox and acetylation states can be summarized in Figure 2.

The role of nutrients can be expanded to inhibit HATs by disrupting their activity. Several HAT inhibitors have been identified and synthesized, targeting HATs to mitigate inflammation and AS. These inhibitors have been found to play a role in histone acetylation regulating genes associated with AS progression, such as early growth response protein 1 (EGR1) [53,54]. However, there is limited information available on exploring and developing nutrients for such purposes, which needs further investigation in the future.

### 3.1. Roles of Nutrients in Regulating Inflammatory Responses

Inflammation, a key characteristic of the innate immune response, can be heightened by the production and translocation of gut-derived lipopolysaccharides (LPS) into the bloodstream. This process triggers the activation of toll-like receptor 4 (TLR4) and NF-κB, which in turn enhances the release of pro-inflammatory cytokines [55]. The production of these cytokines, along with the activation of mitogen-activated protein kinase (MAPK) cascades, such as extracellular signal-regulated kinases (ERKs), and c-Jun N-terminal kinases (JNKs) is initiated by the transforming growth factor β-activated kinase 1 (TAK1) [56]. TLRs activate these signaling pathways by recruiting suitable adaptors, stimulating the generation of pro-inflammatory cytokines. These inflammatory cascades have been closely associated with AS progression, as pattern recognition receptors, such as TLR4, recognize LDLs [55,57]. Pro-inflammatory M1 macrophages play a crucial role in the progression of AS [58], as indicated by their increased presence in atherosclerotic plaques. This suggests that anti-inflammatory agents could be targeted for therapy. The expression of T helper cell 1 (Th1) cytokines, such as TNFα, IL-6, and IL-8, is attributed to the differentiation of M1 macrophages [59]. Additionally, activated platelets release platelet factor 4 (PF4), inducing monocyte differentiation and increasing oxidized LDL uptake by macrophages, which in turn facilitates foam cell formation [60]. These findings support the idea that AS progression can be mitigated by shifting the macrophage phenotype from M1 to M2 [61].

Nutrients play a critical role in preventing inflammatory signaling pathways by effectively inhibiting pro-inflammatory cytokines and blocking downstream inflammatory routes and cascades. The selective functions of nutrients in regulating inflammation stem from their ability to activate SIRTs, such as SIRT1 and SIRT3, while inhibiting classical HDACs [3]. For instance, astaxanthin (ASTX), xanthophyll carotenoid, significantly decreases the expression of pro-inflammatory genes such as IL-1β, IL-6, as well as TNFα secretion and cytosolic and nuclear NF-κB p65 levels in macrophages. This effect is achieved by preventing inflammation through SIRT1 activation [62]. Additionally, ASTX inhibits HDAC4, a class IIa HDAC, which is involved in promoting inflammation due to excessive alcohol [63]. Dietary stress is known to generate an abundance of electrons, ROS, and reactive nitrogen species (RNS), creating an oxidative environment that triggers inflammation [51]. Nutrient-associated enzymes, such as SIRT3, a class III HDAC, have been reported to activate antioxidant genes such as manganese SOD2 and catalase through FOXO3 deacetylation, thereby reducing ROS levels [64]. Evidence suggests that nutrients, including resveratrol and ginsenosides, prevent the development of vascular diseases and reduce the risk of AS by regulating EC metabolism, lipid metabolism, and angiogenesis through SIRT3 activation [47].

Recent studies have demonstrated that the interaction between histone deacetylases, such as SIRT1 and HDAC4, plays a crucial role in regulating inflammation and metabolism [65]. Resveratrol, an activator of SIRT1, impedes gluconeogenesis in insulin-resistant hepatocytes by translocating HDAC4 to the cytoplasm from the nucleus, thereby inactivating it [66]. Moreover, HDAC4 upregulation leads to SIRT1 downregulation during the activation of hepatic stellate cells [67] and in skeletal muscle cells stimulated by interferon γ (IFNγ) [68]. Given that SIRT1 and HDAC4 are involved in modulating inflammation, their interplay has been reported to affect alcohol-induced inflammation in macrophages. For example, ASTX, a nutrient, inhibits alcohol-triggered inflammation and oxidative stress in macrophages by mediating the opposing actions of SIRT1 and HDAC4 [63]. Selected nutrients, such as resveratrol, activate SIRT3. The deacetylation ability of SIRT3 promotes various antioxidant activities, including SOD2 and catalase. These antioxidants help to maintain the oxidant/antioxidant balance and alleviate vascular endothelial oxidative stress [3].

### 3.2. Roles of Nutrients in Regulating Histone Acetylation State

Histone acetylation is carried out by HATs, which transfer an acetyl group (CH_3_CO-) from acetyl-CoA to the lysine-rich histone tails. This process is mediated by bromodomain proteins. In mammalian cells, HATs can be classified into three categories: Gcn5-related N-acetyltransferases (GNAT), MYST, and CBP/p300 [13]. Histone deacetylation, on the other hand, is the reverse process and is carried out by HDACs [22]. Histone acetylation and deacetylation are reversible epigenetic post-translational modifications that are responsive to micro-environmental changes and susceptible to a wide range of modifications [13,22]. As a result, the roles and features of these histone modifications can be employed and utilized by dietary nutrients to regulate inflammatory dysfunctions, such as VI and AS. It has been understood that HATs can be activated by dietary stress stemming from high-fat, high-glycemic diets, or excessive alcohol consumption, as this stress stimulates genes and enzymes sensitive to redox sensing [51]. Nutrients can activate or repress the activity of HDACs through complex mechanisms that depend on their unique structures, functions, and localizations.

Acetyl CoA plays a crucial role in both histone acetylation and de novo lipid synthesis, demonstrating significant effects on histone acetylation and lipid metabolism [69]. Acetyl-CoA can easily enter the nucleus through nuclear pores and acetylate histones, as well as supply acetyl groups for acetylation in the cytoplasm by HAT [70]. Regulating acetyl-CoA levels is essential for controlling the acetylation state of target genes, which in turn affects a series of processes leading to VI and AS. Acetyl-CoA, an acetyl group bound to a cysteine residue of coenzyme A through a thioester bonding, is produced from the conversion of pyruvate by the pyruvate dehydrogenase (PDH) complex in the inner mitochondrial membrane [71]. Excessive ethanol has been reported to significantly decrease PDH activity and inhibit the conversion of pyruvate to acetyl-CoA in macrophages, which leads to an increase in glycolysis rather than the tricarboxylic acid (TCA) cycle. The exceptional increase in glycolysis caused by ethanol can be counteracted by nicotinamide riboside, a nutrient, to restore normal glucose metabolism and energy production [72].

Specific nutrients such as butyrate, ginsenosides, and sulforaphane, known to inhibit class I HDACs such as HDAC1, HDAC2, and HDAC3, may help prevent VI and AS. HDAC1 activation has been associated with a decrease in global H3K9 acetylation in the aortas of apolipoprotein E (ApoE) knockout mice treated with a high methionine diet to induce hyperhomocysteinemia, promoting lipid accumulation in foam cells [73]. HDAC2 overexpression in human aortic ECs suppresses arginase 2 (Arg2) expression, indicating that HDAC2 regulates Arg2 by directly binding to the Arg2 gene promoter. Preventing oxidized LDL-induced HDAC2 downregulation and Arg2 upregulation improves endothelial function [20], suggesting that HDAC2 could be a novel therapy for inhibiting endothelial dysfunction and AS. HDAC3 activates protein kinase B (PKB or Akt) phosphorylation and activity, regulating EC survival during AS development in response to disturbed hemodynamic forces [74]. In macrophages, HDAC3 inhibition promotes an athero-protective phenotype through histone acetylation, accompanied by the gene expression of efflux transporters ATP-binding cassette transporter A1 (ABCA1) and ABCG1, and increases anti-inflammatory and anti-apoptotic capacities [7]. HDAC3 is upregulated in ruptured human atherosclerotic plaques. Furthermore, HDAC3 deletion leads to a pro-fibrotic program via epigenetic regulation of transforming growth factor β1 (TGFβ1), allowing VSMCs to produce collagen and stabilize plaques [75]. This suggests that macrophage specific deletion of HDAC3 could prevent AS development. The efficacy of HDACs in treating VI and AS may depend on the target genes in tissues and organs. For example, HDAC3 deletion in monocytes and macrophages was beneficial for a mouse model associated with AS [76], while blocking HDAC3 decreased cell survival and increased plaque formation in ECs [74]. A single nucleotide polymorphism (rs3791398) in HDAC4 has been reported to associate with carotid intima-media thickness [77].

The function of nutrients to modulate the acetylation state of target genes and control their expression and activity can be influenced by the microenvironments of cells and tissues. Various nutrients, which can activate class III HDACs such as SIRT1 and SIRT3, are known to shift the oxidative condition towards a reductive microenvironment by scavenging ROS and electrons generated by dietary stress, in addition to deacetylating target genes. SIRT1, which is primarily localized in the nucleus, has been shown to have potent cardiovascular and metabolic protective functions [78]. Target substrates of SIRT1 include histones such as acetylated H3K9 and H3K56, as well as non-histone proteins such as NF-κB, FOXOs, p53, PGC-1α, LXRα, and several DNA damage repair proteins including Ku70 and DNA-dependent protein kinase (DNA-PK) [26]. SIRT1 inhibits vascular inflammation, endothelial dysfunction, VSMC proliferation and migration, ROS production, foam cell development, and impaired autophagy, thereby preventing vascular aging, intimal hyperplasia, and AS [79]. Activation of SIRT1 by resveratrol has been shown to attenuate arterial rigidity caused by a high-fat, high-sucrose diet by inhibiting NF-κB-dependent VCAM-1 expression and vascular oxidative stress [80]. SIRT1 inhibits angiotensin II-induced VSMC hypertrophy [81] and migration of VSMC-derived foam cells by oxidized LDL [82], suggesting an overall athero-protective role for SIRT1. SIRT3, exclusively localized in mitochondria, defends against oxidative stress through the direct deacetylation and activation of Mn-SOD. In addition to histone deacetylation, SIRT3 is involved in various mitochondrial functions, including mitochondrial biogenesis, autophagy, and tissue homeostasis, specifically during stressful circumstances [47]. SIRT3 deficiency has been shown to promote thrombus formation by increasing the generation of neutrophil extracellular traps in a carotid thrombosis model stimulated by laser and LPS. A reduction in SIRT3 expression has been observed in leukocytes from individuals with myocardial infarction [83], suggesting that SIRT3 may protect against thrombotic complications associated with myocardial infarction.

## 4. Epigenetic Roles of Nutrients in Regulating VI and AS

### 4.1. Astaxanthin (ASTX)

ASTX, a xanthophyll carotenoid that is soluble in lipids, exhibits anti-inflammatory and antioxidant properties [84]. Its configuration with an oxo functional group enables it to easily traverse the membrane bilayer, without inducing any detrimental side effects [85]. The anti-inflammatory and antioxidant properties of ASTX under dietary stress conditions may be attributed to the activation of SIRT1 and nuclear factor erythroid 2–related factor 2 (NRF2), as well as the enhancement of cellular NAD+ levels [62]. Dietary stress from high-fat or alcohol diets can cause acetylation of NF-κB lysine residues, releasing it from inhibitor of NF-κB (IκB) and allowing nuclear translocation, DNA binding, and transcription of NF-κB target inflammatory genes [86]. The inhibitory effects of ASTX on NF-κB transcription include blocking NF-κB nuclear translocation by deacetylating it [87] and counteracting it by enhancing its co-repressors, such as the transducin-like enhancer of split 1 (TLE1) via SIRT1 activation [88]. Additionally, SIRT1 inactivates NF-κB co-activators such as p300/CBP, preventing the acetylation of p65 NF-κB in the nucleus [89]. Dietary stress stimulates p300/CBP proteins, HATs, to acetylate poly [ADP-ribose] polymerase 1 (PARP-1) and directly interact with p300 for NF-κB activation, which is inhibited by SIRT1 via PARP-1 deacetylation [90].

The ability of ASTX to activate PPARα and repress PPARγ and Akt reduces lipid accumulation in the liver caused by a high-fat diet in mice [91]. The repression of Akt activity by ASTX diminishes nuclear translocation of sterol regulatory element-binding protein 1 (SREBP-1), reducing hepatic lipogenesis. In addition, ASTX induces hepatic autophagy by enhancing PPARα and suppressing PPARγ and Akt [92]. The removal of lipid accumulation by ASTX, which helps to inhibit the progress of VI and AS, can be attributed to the role of SIRT1 in deacetylating functional proteins involved in each pathway.

### 4.2. Butyrate

Butyrate is a short-chain fatty acid found in butter and cheese, formed from dietary fibers by colonic microbiota through fermentation [93,94]. The characteristics of gut microbiota, including structure, diversity, and composition, influence the formation of butyrate, which is mediated by pathological pathways including inflammatory and metabolic diseases such as diabetes and AS [95]. Butyrate is known to mitigate inflammation and prevent cancer, as it mediates energy metabolism and maintains intestinal homeostasis [96]. Butyrate has been reported as a potent inhibitor of primarily class I HDACs, such as HDAC1, 2, 3, and 8, exhibiting anti-inflammatory effects [97]. It inhibits NF-κB activity and TNFα production in LPS-stimulated peripheral blood mononuclear cells (PBMCs) [98] and blocks NF-κB activation in lamina propria macrophages from colitis patients [99]. Butyrate suppresses HDAC3 activity in the liver, inducing fibroblast growth factor 21 (FGF21) to enhance fatty acid oxidation, ketogenesis, PGC-1α expression, and the flow of the TCA cycle [100,101]. The impact of butyrate on drug metabolism may be linked to its ability to hinder HDACs through epigenetic actions [24]. By inhibiting HDAC activity, butyrate demonstrates protective characteristics against glucose intolerance and insulin resistance caused by a high-fat diet [96]. By suppressing HDAC activity, it stimulates NRF2 activation in IEC-6 cells of the small intestine and human colorectal adenocarcinoma (HT-29 cells) [102].

### 4.3. Curcumin

Curcumin, a polyphenolic curcuminoid from Curcuma longa L. (turmeric) rhizome, is widely used as a spice ingredient in Asian countries [103]. In addition to its well-known anti-inflammatory and antioxidant effects [104], curcumin has demonstrated anti-carcinogenic and neuroprotective properties [105] helping to protect against cardiovascular disease and endothelial dysfunction [106]. Curcumin has been shown to increase flow-mediated dilation, a measure of endothelial function, improving endothelial function and NO bioavailability associated with reduced vascular oxidative stress, in both normal individuals [107] and those at risk of cardiovascular disease [108]. Obese rats fed a high-fat, high-sugar diet with curcumin exhibited lower levels of inflammatory biomarker C-reactive protein (CRP), adhesion molecules (VCAM-1 and ICAM-1), but higher levels of NO metabolites and antioxidant enzyme catalase activity compared to the control group [109,110]. Considering that adhesion molecules are associated with inflammation, these findings indicate that curcumin could prevent vascular damage by decreasing inflammation and vascular oxidative damage in rats fed a high-fat and high-sugar diet [110].

Excessive cholesterol absorption in the intestine can lead to elevated plasma cholesterol levels, promoting the development of AS [111]. Curcumin has been reported to reduce intestinal cholesterol absorption and prevent AS in high-fat diet-fed ApoE knockout mice, indicating a potent anti-atherogenic action [112]. Curcumin reduced pro-inflammatory cytokines including IL-1β, IL-6, and TNFα, as well as M1 cell apoptosis-induced oxidized LDL, while upregulating cluster of differentiation 36 (CD36) and ABCA1 in M1 macrophages [113]. The capacity of M1 macrophages to promote lipid processing, disposal, and elimination, supporting cholesterol homeostasis and exerting a protective effect against AS, could be improved by curcumin [113]. Curcumin also ameliorated oxidized LDL-induced damage in human umbilical vein ECs by targeting microRNA-599 (miR-599) to regulate myeloid differentiation primary response 88 (MYD88) expression, and deactivating the NF-κB pathway in a cell model of AS [114]. Despite being insoluble in water, curcumin can withstand the acidic conditions of stomach and regulate gut functions [103]. Curcumin attenuated cadmium-induced AS by inhibiting the intestinal flora imbalance, increasing trimethylamine-N-oxide synthesis, and macrophage polarization through remodeling the gut microbiota [115].

In hypoxic cells, including those found in AS and cancer, curcumin exhibits HAT inhibitory effects by inducing apoptosis and inhibiting p300/CBP through p53 signaling [116]. Curcumin can also prevent an increase in acetylation levels resulting from dietary factors and mitigate insulin resistance associated with diabetes through the inhibition of eNOS by suppressing p300/CBP and NF-κB [117,118]. Within foam cells derived from macrophages, curcumin upregulates the expression of ABCA1 by enhancing the AMPK-SIRT1-LXRα signaling pathway to enhance cholesterol efflux [119]. The delay in lipid accumulation that forms foam cells, leading to AS, can be attributed to SIRT1 activation [29].

### 4.4. Ginsenoside

The active constituents of ginseng, known as ginsenosides, exhibit a broad spectrum of biological and medicinal effects, which include anti-inflammatory and antioxidant properties [120]. Compounds such as ginsenosides Rb1 and Rg3, compound K, as well as red ginseng extract exert anti-inflammatory effects on macrophages through the NF-κB or MAPK pathway [121,122,123,124]. Ginsenoside F1 has been shown to protect against EC damage and prevent AS progression in ApoE knockout mice by inhibiting LOX-1 and NF-κB [125]. In addition, ginsenoside Rg3 reverses endothelial-to-mesenchymal transition by regulating the miR-139-5p- NF-κB axis, to avoid vascular disease [126]. Ginsenosides Rg1 and Rb1 prevent monocyte adhesion in human coronary artery ECs by inhibiting TNFα-induced ICAM-1 and VCAM-1 expression and preventing NF-κB translocation into the nuclei of artery ECs [127]. Rg3-enriched Korean red ginseng enhances eNOS levels while suppressing TNFα-mediated ICAM-1 and COX-2 in human umbilical vein ECs, improving vascular function in spontaneously hypertensive rats [128]. Ginsenoside Rb1 has also been demonstrated to inhibit AS progression and reduce serum MCP-1 expression in ApoE knockout mice [129]. Ginsenoside Rk1 impedes platelet aggregation, granule secretion, calcium ion mobilization, and integrin αIIbβ3 activation, while increasing cAMP levels [130]. These findings highlighted the roles of ginsenosides in immune responses related to AS and platelet aggregation [131]. Ginsenoside Rg2, Rg3, and the protopanaxatriol fraction exhibit potent effects in inhibiting clotting factors such as coagulation factor Xa (FXa) [132]. Both ginsenosides Rg1 and Rg2 exhibit anti-coagulatory activities by prolonged clotting time [133]. In rats fed a high-fat diet, ginseng berry extract has been shown to modulate the metabolism of lipids and blood coagulation factors [134], implying that ginsenosides may be an effective treatment for AS resulting from high-fat diet-induced blood coagulation.

The functions of ginsenosides have been linked to histone modifications, such as acetylation and deacetylation [135]. Ginsenoside compound K suppresses HDAC1 expression, regulating histone H3 and H4 acetylation states to impede the proliferation and induce apoptosis in HT-29 cells [136]. Ginsenoside 20(s)-Rh2 inhibits the growth of human leukemia cells by inhibiting HDAC1, HDAC2, and HDAC6 [137], while ginsenoside Rg3 impedes melanoma cell proliferation by reducing HDAC3 expression [138]. Emerging potential applications for ginsenosides include activating SIRT1 to regulate inflammation, oxidative stress, and metabolic dysfunction [51]. Studies have confirmed that ginsenosides elevate NAD+ levels in metabolically active tissues, including skeletal muscle, brown adipose tissue, and liver, stimulating mitochondrial biogenesis and oxidative phosphorylation [120]. This protective effect against metabolic diseases may also extend to VI and AS.

### 4.5. Nicotinamide Riboside (NR)

NR is a natural precursor of NAD+ and a component of pro-vitamin B3 found in cow milk [139]. NR activates SIRT1 by providing the cofactor, NAD+, which is essential for modulating cellular processes, including metabolic pathways [140]. NR demonstrates anti-inflammatory and antioxidant effects, mitigating metabolic disturbances in macrophages caused by dietary stress through SIRT1 activation [141]. NR’s ability to activate SIRT1 influences energy metabolism, including alterations in glycolysis and mitochondrial respiration [72,142]. Inflammatory conditions facilitated by dietary stress lead to HIF-1α activation, which was counteracted by NR supplementation through deacetylation of HIF-1α by SIRT1 [143]. This repression affects downstream genes, such as glucose transporter 1 (GLUT1), pyruvate dehydrogenase kinase 1 (PDK1), and lactate dehydrogenase α (LDHα) [72,144]. Lactate-induced enhancement of HIF-1α increases glycolysis and suppresses the TCA cycle through the activation of PDK1 [145,146]. Pyruvate, the final product of glycolysis, is transformed into acetyl-CoA inside the mitochondria by the action of PDH. PDK1 inhibits this conversion by phosphorylating PDH [71]. Since SIRT1 inhibits the expression GLUT1, PDK1, and LDHα, NR can alleviate metabolic disturbances induced by dietary stress through SIRT1 activation due to its deacetylation ability [72,142]. Furthermore, NR modulates mitochondrial biogenesis in hepatocytes via SIRT1-mediated deacetylation of PGC-1α [142,147].

### 4.6. Quercetin

Quercetin, a dietary flavonoid present in various foods derived from plants, such as red onions, tea, broccoli, apples, capers, parsley, berries, and red grapes [148], exhibits anti-inflammatory and antioxidant properties [149]. Its inhibitory effects on high fructose-induced AS have been demonstrated through the reduction of ROS levels, caspase-3 activation, inflammatory cytokine levels, collagen content, and the expression of inflammation-related proteins [150]. Quercetin also protects against AS by hindering plaque development in high fructose-fed mice through the regulation of PI3K/Akt activation by ROS [150]. Its ability to reduce the atherosclerotic plaque area can be attributed to the attenuation of oxidative stress, reduced aortic p47 and p67phox expressions, and partial reversal of NOX4 (NADPH subunit) expression compared to the high-fat diet-fed ApoE knockout mice group [151]. Furthermore, in mouse peritoneal macrophages, quercetin inhibits oxidized LDL-induced ROS formation and blocks the activation of NADPH oxidase by preventing the membrane translocation of p47phox [151].

Quercetin’s competitive binding to kelch-like ECH-associated protein 1 (KEAP1) at Arg483 is suggested as an anti-atherosclerotic mechanism that inhibits macrophage pyroptosis [152]. Quercetin suppresses oxidized LDL-induced pyroptosis in THP-1 derived macrophages at the cellular level by decreasing the NLR family pyrin domain-containing 3 (NLRP3) inflammasome activation and ROS levels, which were reversed by the specific NRF2 inhibitor, ML385 [152]. Quercetin promotes NRF2 dissociation from KEAP1, enhancing NRF2 nuclear translocation and transcription of downstream antioxidant enzymes [153]. Quercetin also inhibits oxidized LDL-induced senescence in plaque macrophages by suppressing the p38MAPK/p16 pathway [154]. Vascular disturbed flow is recognized as a key factor leading to EC dysfunction. The ability of quercetin to protect endothelial function against inflammation resulting from localized disturbed flow involves suppressing neuropilin-2 (NRP2) in human umbilical vein ECs [155]. Moreover, quercetin defends against oxidized LDL-induced endothelial oxidative damage by maintaining mitochondrial function, inhibiting ROS formation, and suppressing NF-κB signaling through the epigenetic activation of SIRT1 [156].

### 4.7. Resveratrol (RES)

RES, a natural polyphenol primarily found in grapevines, has demonstrated anti-atherosclerotic effects in cardiovascular cells as well as atherosclerotic ApoE knockout mice [157]. RES has been demonstrated to inhibit the TGF/ERK signaling pathway, which is involved in VSMC proliferation and inflammation, and is related to AS development [158]. The epigenetic activation of SIRT1 and SIRT3 by RES contributes to its anti-atherosclerotic potential [159]. RES inhibits the TGFβ/suppressor of mothers against decapentaplegic (Smad2/3) pathway by activating SIRT1, inhibiting TGFβ receptor type I (TβRI) and phospho-Smad2/3 expression, and blocking Smad2/3 nuclear translocation [160]. In addition, RES represses transcription factor Smad3 activity through SIRT3 activation [161]. By downregulating miR-17, RES promotes Smad7 expression, resulting in the repression of the TGFβ1/Smad signaling pathway [162]. Moreover, RES inhibits TGFβ-mediated ERK signaling pathway, further repressing the TGF/ERK pathway [158].

RES has been shown to regulate lipid metabolism, inflammation, and oxidative stress, inhibiting AS development at various stages [159]. It also exhibits multiple beneficial functions against AS such as anti-aging, immunoregulation, and cardioprotection, and modulates vascular homeostasis, intestinal microbiota, lipoprotein oxidation, monocyte adhesion, platelet aggregation, and thrombosis [163]. RES has been found to inhibit AS lesions, myocardial infarction, heart failure, and ischemia/reperfusion injuries while improving plaque stability [164]. It suppresses the accumulation of lipids in macrophages, inhibiting the formation of foam cells induced by LPS [165], reduces thrombosis and lipid accumulation to prevent AS development, and inhibits diet-induced cardiac abnormalities [166] and vascular injury-induced inflammasome activation [167] through SIRT1 activation.

RES promotes the transition of VSMC phenotype from contractile to differentiation state by stimulating SIRT1 and AMPK [168]. The role of SIRT1 in deacetylating the RelA/p65 subunit at lysine 310 represses NF-κB. RES reduces TNFα-induced ICAM-1 activation, inhibiting inflammation [169]. RES has been found to induce autophagy via SIRT1 activation, attenuating TNF-triggered inflammation in ECs [170]. Furthermore, RES therapy affects the association between SIRT1 and NRF2. SIRT1 triggers the activation of NRF2 by lowering its acetylation levels in response to oxidative stress, indicating that the antioxidant properties of NRF depend on its acetylation state [171]. RES has been shown to activate SIRT1, promoting translocation of NRF2 into the nucleus and its transcriptional activity, resulting in NRF2-controlled gene expression [171].

### 4.8. Sulforaphane (SFN)

SFN is an isothiocyanate compound present in cruciferous vegetables such as broccoli and kale, known to trigger phase II detoxifying/antioxidant enzymes through the activation of NRF2 [172]. Demonstrating antioxidant and anti-inflammatory effects, SFN activates NRF2 under oxidative stress conditions, and suppresses cancer cell growth, including HCT116 human colorectal carcinoma cells, by inhibiting HDAC2 and HDAC3 activities [173,174]. SFN is involved in epigenetic pathways as an HDAC inhibitor, affecting the expression of key HDACs and HATs, causing genome-wide epigenetic alterations [175]. Recent studies suggest the combinational epigenetic mechanisms of SFN with butyrate in inhibiting breast cancer [176], which could be utilized for AS prevention due to similarities in local hypoxia conditions between atherosclerotic plaque and cancer [29]. SFN metabolites, such as SFN-N-acetyl-cysteine, likely increase histone H3 and H4 acetylation states, inhibiting HDACs in colon cancer cells [177,178]. SFN can suppress the activation of NF-κB [179] and promote cell death through the activation of caspases by inhibiting HDAC6 activity, which increases the acetylation states of p21 and Bcl-2-associated X protein (BAX) promoters [180]. In prostate cancer cells, SFN inhibits HDAC6 activity, increasing the level of acetylation in heat shock protein 90 (Hsp90) and decreasing androgen receptor (AR) expression [181]. SFN reduces HDAC1, 4, 5, and 7 protein levels in cancer cells [173] and inhibits class I HDACs, such as HDAC2, as well as class IIa HDACs, such as HDAC4, 5, 7, and 9, in 3T3-L1 adipocytes [174]. SFN elevates histone H3 acetylation levels in TRAMP-C1 cells, mouse prostate epithelial cells, increasing the activity of NRF2 and inducing its expression [181]. Recently, studies have indicated that SFN can inhibit cell cycle arrest and apoptosis in oral cancer cells by repressing HDACs [173].

## 5. Conclusions

The review focuses on the potential of certain nutrients to prevent VI and AS by regulating acetylation states. AS is a metabolic-driven chronic disorder triggered by inflammation and oxidative stress. However, it can be prevented by selected nutrients that counteract the detrimental conditions arising from high cholesterol or triglyceride levels in the vascular environment. Nutrients have two ways of preventing VI and AS: scavenging reactive radicals such as ROS due to their chemical structure, and regulating immunity systems and gene expression by modulating target proteins, which alter the acetylation state. Epigenetic histone modifications are associated with HATs and HDACs at the post-translational level, and their interactions depend on the conditions of cells or tissues, particularly in pathogenesis and stress. Selected nutrients can impact the activities of HATs and HDACs to regulate gene activation or repression, with the help of related genes. In general, selected nutrients repress class I and II HDACs, while activating class III HDACs, and inhibit HATs that generate oxidative stress and inflammation, leading to VI and AS. However, there remain gaps in the knowledge that need to be addressed for a more effective programmed approach by using nutrients to treat multiple risks leading to AS simultaneously and harmoniously. Specifically, schemes for enhancing the bioavailability of nutrients and understanding their specific roles depending on the target genes need to be investigated. Further research is required to elucidate the roles of nutrients involved in the interactions between HATs and HDACs, with a particular focus on their specific effects on both HATs and HDACs, in order to enhance the understanding of nutrient usage.

## Data Availability

Not applicable.
